# Metabolic States Following Accumulation of Intracellular Aggregates: Implications for Neurodegenerative Diseases

**DOI:** 10.1371/journal.pone.0063822

**Published:** 2013-05-07

**Authors:** Alexei Vazquez

**Affiliations:** Department of Radiation Oncology and Center for Systems Biology, The Cancer Institute of New Jersey, Robert Wood Johnson Medical School, University of Medicine and Dentistry of New Jersey, New Brunswick, New Jersey, United States of America; University of Rostock, Germany

## Abstract

The formation of intracellular aggregates is a common etiology of several neurodegenerative diseases. Mitochondrial defects and oxidative stress has been pointed as the major mechanistic links between the accumulation of intracellular aggregates and cell death. In this work we propose a “metabolic cell death by overcrowding” as an alternative hypothesis. Using a model of neuron metabolism, we predict that as the concentration of protein aggregates increases the neurons transit through three different metabolic phases. The first phase (0–6 mM) corresponds with the normal neuron state, where the neuronal activity is sustained by the oxidative phosphorylation of lactate. The second phase (6–8.6 mM) is characterized by a mixed utilization of lactate and glucose as energy substrates and a switch from ammonia uptake to ammonia release by neurons. In the third phase (8.6–9.3 mM) neurons are predicted to support their energy demands from glycolysis and an alternative pathway for energy generation, involving reactions from serine synthesis, one carbon metabolism and the glycine cleavage system. The model also predicts a decrease in the maximum neuronal capacity for energy generation with increasing the concentration of protein aggregates. Ultimately this maximum capacity becomes zero when the protein aggregates reach a concentration of about 9.3 mM, predicting the cessation of neuronal activity.

## Introduction

The formation of intracellular aggregates is a common etiology of several degenerative diseases [1], [2], [3]. In the context of neurodegenerative diseases, the extracellular and intracellular accumulation of the aggregates of β-amyloid protein has been observed in certain brain areas of Alzheimer's patients [3], α-synuclein in Parkinson's patients [1], [3], and hungtingtin in Huntington's patients [4], [5]. Beyond neurodegenerative diseases, the intracellular accumulation of β-amyloid protein aggregates has been hypothesized as a cause of inclusion-body myositis [6], [7], a muscular degenerative disease that starts after age 50 years. The connection between accumulation of intracellular aggregates and degeneration is supported by *in vitro* studies reporting a negative correlation between the accumulation of intracellular aggregates and cell survival [8], [9].

Mitochondrial defects and oxidative stress has been pointed as the major mechanistic links between the accumulation of intracellular aggregates and cell death [9], [10], [11]. Impaired mitochondrial biogenesis contributes to mitochondrial dysfunction in *in vitro* models of Alzheimer's disease [12]. Mouse models of familial Alzheimer's disease have provided evidence indicating that defects in mitochondria trafficking and integrity precede the onset of neurological phenotype [13]. They have also shown that the distribution of mitochondria is disrupted by the formation of protein aggregates in neurons[14]. Taking together this evidence points to reduced mitochondrial activity as a major factor in Alzheimer's disease.

The progressive increase of the intracellular concentration of protein aggregates may also constraint the intracellular space available to metabolic enzymes and mitochondria. The cell has a high density of macromolecules occupying about 30–40% of the intracellular space [15], [16]. An increase of the macromolecular density beyond this value hinders dramatically the diffusion of metabolites and macromolecules, resulting in an exponential reduction of the rate of diffusion-limited reactions [17], [18]. The cell molecular machinery thus operates under a macromolecular crowding constraint, whereby the concentration of macromolecules should not exceed the limiting value of about 40%. However, the impact of molecular crowding on neuron metabolism has remained unexplored.

Brain metabolism has been studied extensively using different experimental techniques. Time resolved experiments have shown that the early neuronal activity (up to about 10 sec) is supported by oxidative phosphorylation of lactate from an extracellular pool [19], [20], [21]. This initial phase is followed by the activation of the astrocyte-neuron lactate shuttle, whereby astrocytic glycolysis generates lactate that is then utilized by the neurons [19], [20], [21]. This evidence indicates that normal neurons undergoing minor neural activity satisfy their energy demands through the oxidative phosphorylation of lactate. However, high lactate levels have been observed in brain regions of Huntington's disease patients compared to normal controls [22], [23], suggesting a shift from a net consumption to a net lactate production by the astrocyte-neuron system. It has also been observed that healthy, mature, non-starved brains take up ammonia [24] while the brains of Alzheimer patients release ammonia [24], [25].

In this work we provide evidence that these altered metabolic phenotypes could be the consequence of an increase in molecular crowding due to accumulation of protein aggregates.

## Results

### *In silico* model of human cell metabolism

To investigate the impact of protein aggregates accumulation on cell metabolism we use an *in silico* model of human cell metabolism [26]. The model is based on a genome-scale reconstruction of the metabolic network of a generic human cell [27]. One could argue that this model should be constrained to take into account the specific pathways that are known to be active in neurons, e.g. by inspecting gene expression data to determine the presence/absence of genes coding for metabolic enzymes [28], [29], [30]. However, we take a different approach. We hypothesize that the specific metabolic pathways that are active in neurons are those satisfying the neuron metabolic demands with the minimal use of nutrients. Therefore, our model aims to predict rather that impose which metabolic pathways are active/inactive.

We will utilize energy demand (moles of ATP consumed per unit time and cell volume) as a surrogate for neuronal activity. The scope of the model is to predict which metabolic pathways satisfy this energy demand with the minimal use of nutrients. We note that resting human cells require energy to maintain their basal functions, which has been estimated to be about 5 mM ATP/min [31]. Most of the results reported below are obtained assuming this energy demand, but we will also investigate scenarios where the energy demand increases beyond the basal value during increased neuronal activity. In addition we take into account that there is a basal rate of RNA and protein turnover and a corresponding basal rate of RNA and protein synthesis.

In this work we focus on stationary metabolic states of neuronal activity. This is a reasonable approximation for resting neurons with a steady energy demand for cell maintenance. A stationary state approximation also applies to modeling sustained neuronal activity with a steady energy demand. Under the steady state approximation the production and consumption of every metabolite balances and the metabolite concentrations remain constant (flux balance constraint). There are of course other physiological regimes that are dynamic in nature. However, we will show that even within the context of the steady state approximation we obtain very interesting predictions regarding the differential utilization of metabolic pathways.

For any given stationary distribution of metabolic fluxes there is a corresponding distribution of metabolic enzymes, ribosomes and mitochodria that is required to catalyze those reactions. The total volume occupied by these macromolecules/organelles should not exceed 40%, which is the typical macromolecular volume fraction in human cells (molecular crowding constraint). In addition, we take into account the volume fraction occupied by non-metabolic macromolecules, including structural proteins and protein aggregates. In a normal state, most of the cellular proteome is composed of structural proteins (e.g., actin) and macromolecules/organelles necessary to maintain their basal metabolism. The concentration of non-metabolic proteins is about 3 mM [26]. The concentration of metabolic enzymes, ribosomes and mitochondria is estimated self-consistently by our model depending on the estimated metabolic fluxes of the corresponding reactions. To model the formation of protein aggregates we simply increase the amount of non-metabolic protein from its basal value of 3 mM.

Summarizing, the metabolic model studied here predicts the metabolic fluxes that will result in a net generation of a predefined ATP demand (e.g., 5 mM ATP/min for cell maintenance) with the minimal use of nutrients and satisfying the flux balance and molecular crowding constraints. Since glucose and lactate are the major energy sources for neurons, we focus on their differential utilization depending on the energy demand and the concentration of protein aggregates. In addition, we assume that the cell media contains all essential nutrients (essential amino acids, ions, etc) and oxygen.

### Exchange fluxes define different metabolic phases

First, we focus on the impact of protein aggregates accumulation on the metabolism of resting neurons. We assume that the metabolic requirements of resting neurons are a basal ATP demand for cell maintenance and basal rates of RNA and protein synthesis to compensate for the basal turnover rates of RNA and protein, respectively.

**Figure 1:**
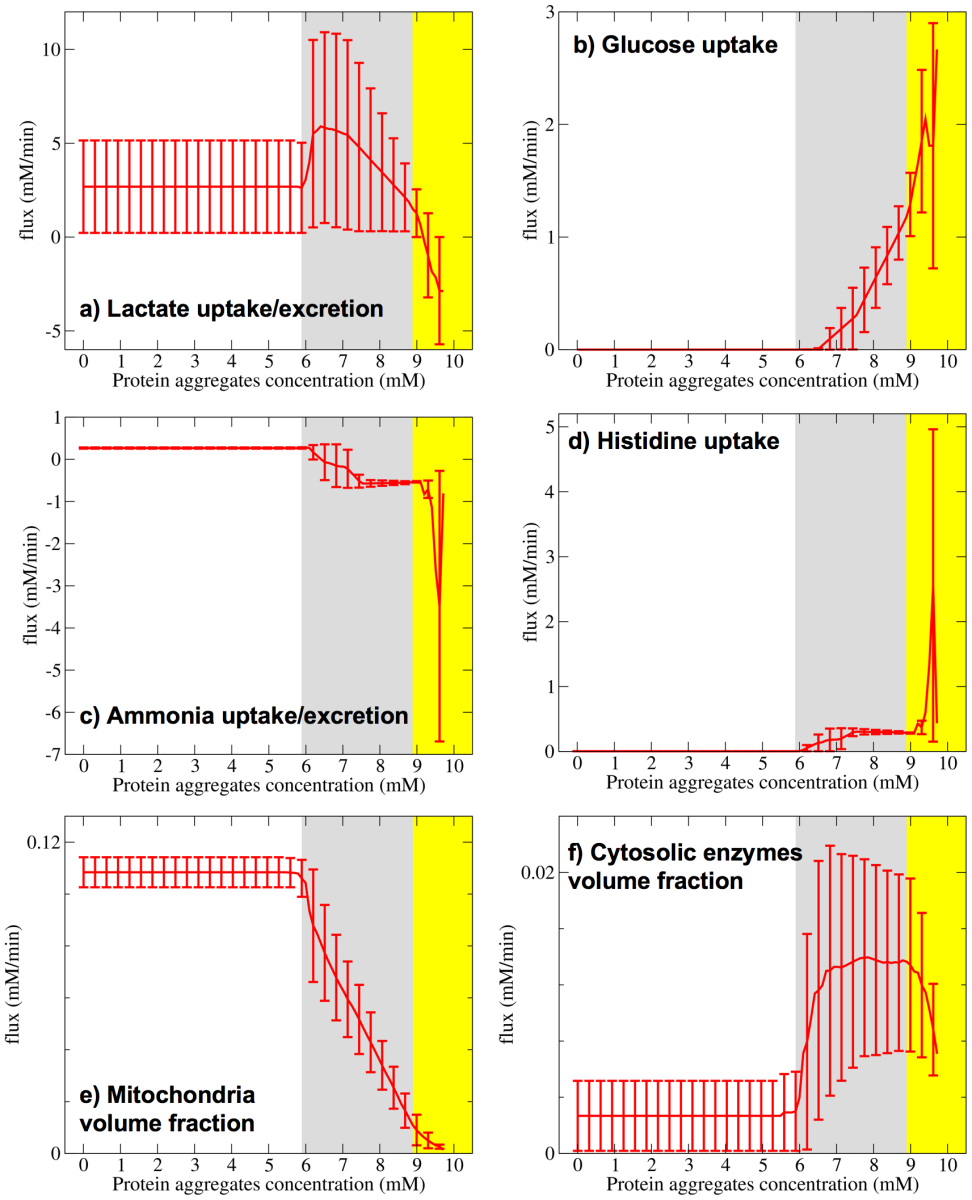
Metabolic phases with increasing the concentration of protein aggregates. a)–d) Exchange flux as a function of the protein aggregates concentration. e) and f) Relative volume fraction occupied by mitochondria e) and cytosolic enzymes f). The lines represent the median over simulated sets of kinetics parameters and the error bars are the 90% confidence intervals. The white, grey and yellow background represent phase 1, 2 and 3, respectively.

We observe significant changes in the model predicted exchange fluxes when increasing the concentration of protein aggregates (Figure 1). Specifically, we can clearly distinguish three major phases depending on whether lactate or glucose is used as the energy substrate and whether ammonia or histidine is used as the nitrogen source. Below protein aggregate concentrations of 6 mM, lactate is the preferred energy substrate and ammonia the preferred nitrogen source (Phase 1, white background). From 6 to 8.6 mM there is a transition to a mixed use of lactate and glucose as energy substrates and a gradual change from ammonia to histidine as nitrogen source, with concomitant excretion of ammonia (Phase 2, grey background). From 8.6 to 9.3 mM there is a complete switch to glucose as the energy substrate and histidine as the nitrogen substrate, with concomitant excretion of lactate and ammonia (Phase 3, yellow background). Finally, beyond 9.3 mM the protein aggregates occupy almost all the 40% of the intracellular volume accessible to macromolecules and there is no room for the allocation of metabolic enzymes, ribosomes or mitochondria (unfeasible region). In this latter regime the cell is predicted to be incapable of generating the basal energy required for cell maintenance and therefore should die.

These changes in exchange fluxes are accompanied by changes in the relative abundance of mitochondria and cytosolic enzymes. For protein aggregate concentrations below 6 mM mitochondria occupy about 12% if the intracellular volume (Figure 1e). However, beyond 6 mM the relative volume fraction occupied by mitochondria gradually decreases, becoming zero at the maximum feasible concentration of protein aggregates. This gradual decrease is accompanied by an increase of the volume faction occupied by cytosolic enzymes (Figure 1f). These changes in volume fractions are a reflection of internal changes in the metabolic flux distribution as discussed below.

### Phase 1: Normal state, lactate utilization

In Phase 1 lactate is the preferred energy substrate (Figure 1a). After uptake the model predicts its conversion to pyruvate, which then fuels the oxidative phosphorylation in the mitochondria. There is also an additional uptake of ammonia and amino acids to sustain the basal rate of RNA and protein synthesis associated with the corresponding basal rates of RNA and protein degradation. This phase agrees with what is reported for normal neurons with minor activity, where energy is mostly generated from oxidative phosphorylation of lactate [19], [20], [21].

### Phase 2: Mixed lactate-glucose utilization

**Figure 2:**
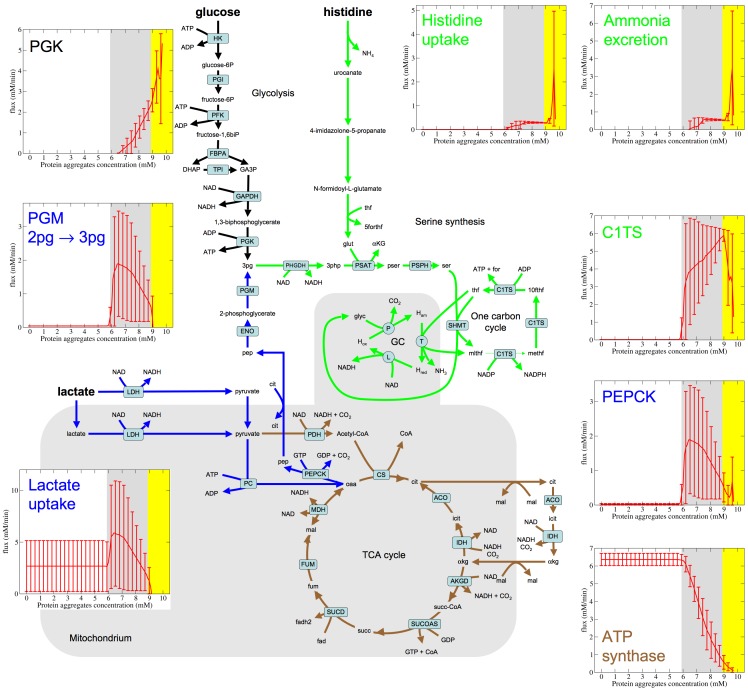
Metabolic flux distribution in Phase 2. Rate of selected reactions that are relevant to the metabolic state of Phase 2. The lines represent the median over simulated sets of kinetics parameters and the error bars are the 90% confidence intervals. The white, grey and yellow backgrounds represent Phase 1, 2 and 3, respectively. Abbreviations: PGK, phosphoglycerate kinase; PGM, phosphoglycerate mutase; PEPCK, phosphoglycerate carboxykinase; C1TS, C1-tetrahydrofolate synthase, a tri-functional enzyme that processes three distinct enzymatic activities, 5,10-methylenetetrahydrofolate (mlthf) dehydrogenase, 5,10-methenyltetrahydrofolate (methf) cyclohydrolase and 10-formyltetrahydrofolate (10fthf) synthetase.

In this phase there is a mixed utilization of lactate and glucose as energy substrates (Figure 1a and 1b). There are also significant changes in the way lactate is metabolized (Figure 2). As in Phase 1, lactate is converted to pyruvate. Part of the pyruvate is used for oxidative phosphorylation in the mitochondria, with a magnitude that gradually decreases with increasing the concentration of protein aggregates (Figure 2, brown). This behavior is quantified by the gradual decrease of the ATP synthase rate (Figure 2, ATP synthase panel), which matches the predicted decrease in mitochondrial density (Figure 1e). The remaining part of pyruvate is converted to 3-phosphoglycerate in a glyconeogenesis like fashion (Figure 2, blue). Glucose also contributes to the production of 3-phosphoglycerate via the first steps of glycolysis. 3-phosphoglycerate is then metabolized through an alternative pathway for energy generation involving reactions from serine synthesis, one carbon metabolism and the glycine cleavage system (SOG pathway, Figure 2, green), first reported in [26]. This pathway generates 4 molecules of ATP per molecule of glucose and 2 molecules of ATP per molecule of lactate. In this phase both the partial oxidative phosphorylation of lactate and the SOG pathway sustain the basal energy demand. The upregulation of the SOG pathway is accompanied by a change from ammonia to histidine as the nitrogen source (Figure 2, histidine uptake panel) and a switch from ammonia uptake to ammonia excretion (Figure 2, ammonia excretion panel).

### Phase 3: Glucose utilization with lactate and ammonia excretion

**Figure 3:**
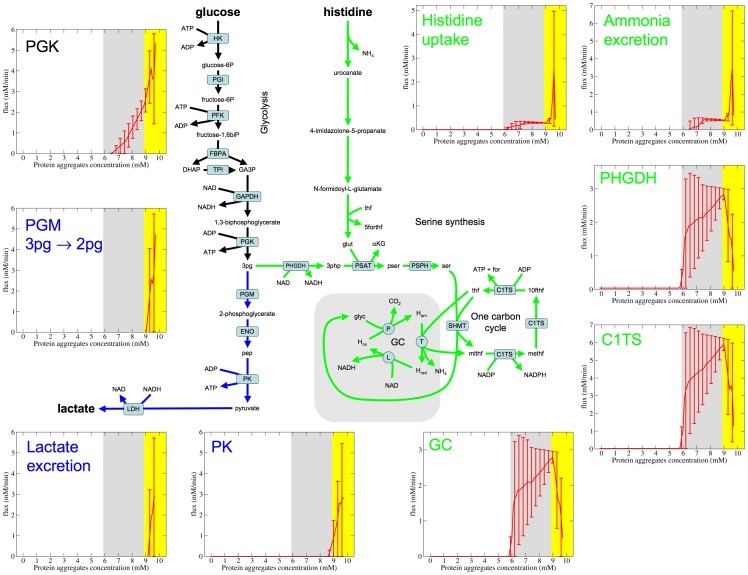
Metabolic flux distribution in Phase 3. Rate of selected reactions that are relevant to the metabolic state of Phase 3. The lines represent the median over simulated sets of kinetics parameters and the error bars are the 90% confidence intervals. The white, grey and yellow backgrounds represent Phase 1, 2 and 3, respectively. Abbreviations not found in Figure 2: PK, pyruvate kinase; PHGDH, phosphoglycerate dehydrogenase; GC, glycine cleavage complex.

In this phase there is a complete switch to glucose as the energy substrate (Figure 3). Glucose is metabolized to 3-phosphoglycerate. 3-phosphoglycerate is then metabolized to either lactate (Figure 3, blue), completing the glycolysis pathway, or through the SOG pathway (Figure 3, green). In this phase histidine is again the nitrogen source (Figure 3, histidine uptake panel) and ammonia is excreted (Figure 3, ammonia excretion panel). We note a large variability in the model predictions due to uncertainties in the model kinetic parameters.

### Phase diagram

**Figure 4:**
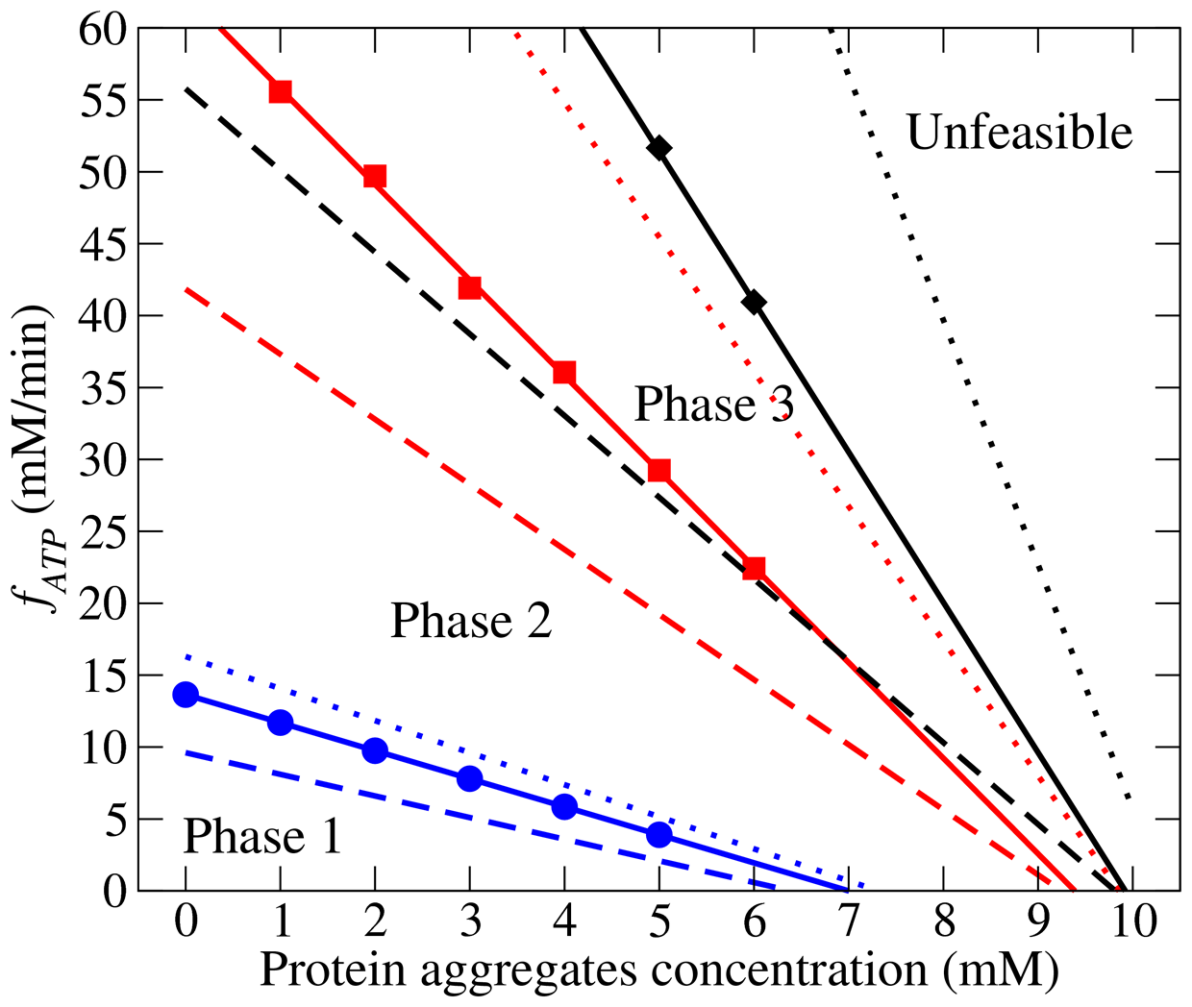
Phase diagram. Predicted metabolic phases as a function of the concentration of protein aggregates and the energy demand. The symbols represent energy demands where, for 50% of the kinetic parameters choices, the glucose uptake starts increasing from zero (circles), the lactate exchange switch from uptake to excretion (squares) and the maximum attainable ATP demand (diamons), as a function of the concentration of protein aggregates. The dashed, solid and dotted lines represent the energy demands where in 5%, 50% and 95% of the kinetic parameter choices the event specified by the corresponding symbol was satisfied, after linear fits to the simulation points.

These results can be generalized to situations where the neuronal activity demands more energy than what is required for cell maintenance. More precisely, we can depict the different metabolic phases in the plane of protein aggregate concentration and energy demand (Figure 4). To this end, we determined the energy demand when glucose uptake starts (Phase 1/Phase 2), when lactate uptake switches to lactate excretion (Phase 2/Phase 3) and the maximum energy demand that can be satisfied (Phase 3/Unfeasible region), all as a function of the concentration of protein aggregates.

The model predicts that the transition from Phase 1 to Phase 2 and from Phase 2 to Phase 3 is shifted to lower protein aggregate concentrations as the energy demand is increased. We also note that the maximum ATP demand that can be satisfied by the cell metabolism decreases as the protein aggregate concentration increases (Figure 4, line demarking phase 3 from the unfeasible region). In other words, the increase of the protein aggregate concentrations results in a gradual decrease of the cell capacity for energy generation.

Finally, we note that the confidence intervals of the transition regions for (Phase 2/Phase 3) and (Phase 3/Unfeasible region) overlap. This observation simple means that for certain choices of kinetic parameters the Phase 3 is not observed.

## Discussion

This *in silico* study leads to very interesting predictions. It indicates that neurons can be in three different metabolic phases depending on the magnitude of the protein aggregate concentrations and the energy demand. Phase 1 represents the normal state of neurons, where the energy requirements are satisfied through oxidative phosphorylation of lactate. The formation of protein aggregates and/or an increase in the energy demand to satisfy neuronal activity can shift the neuron to the Phase 2, characterized by a mixed oxidative phosphorylation of lactate, the production of 3-phosphoglycerate from lactate and glucose, and the metabolism of 3-phsophoglycerate via the SOG pathway, with concomitant histidine uptake and ammonia excretion. A further increase in the protein aggregate concentration and/or a further increase of the energy demand can shift the neuron to Phase 3, where the energy demand is sustained by glycolysis, with concomitant lactate excretion, and the SOG pathway, with concomitant histidine uptake and ammonia excretion.

The contribution of the SOG pathway to energy production is at the current stage theoretical. This pathway has been predicted to be upregulated in highly proliferating cells [26], which are also subject to a high energy demand to fuel biosynthesis of the cell components. Our ongoing experimental work in the context of proliferating cells indicates that the activity of this pathway indeed increases with increasing proliferation. Furthermore, inhibition of the pathway, by knockdown of intermediary stems, or by pharmacological inhibition with antifolates targeting the one-carbon steps, causes energy stress (work in progress). While we cannot warranty that there may be tissue differences, our work in progress is providing support for the involvement of the SOG pathway in energy generation.

The model also predicts that neurons have a maximum capacity for energy generation and that this maximum capacity decreases with increasing the concentration of protein aggregates. The maximum capacity becomes zero at protein aggregate concentrations of about 9.3 mM. Beyond the latter value the cell cannot even satisfy is basal energy demand for cell maintenance and therefore the neuron is predicted to die. We call this predicted cell death “metabolic cell death by overcrowding”. This prediction is supported by empirical data showing a negative correlation between the formation of intracellular aggregates and cell survival in human embryonic kidney cells [8] and in neurons [9]. The metabolic cell death by overcrowding may actually be a general phenomenon in cell biology, since there is a negative correlation between the formation of intracellular aggregates and cell survival in the bacterium *E. coli* as well [32].

Further support for the *in silico* predictions comes from measurements of mitochondrial content and of metabolic byproduct accumulation in the brain of neurodegenerative disease patients. The model prediction of a decrease in mitochondria content as the concentration of protein aggregates increases agrees with the experimental observation of a lower content of mitochondria in the neurons of Alzheimer's [10], [33] and Huntington's [11] patients. Higher lactate levels have been observed in certain brain regions of Huntington's disease patients compared to normal controls [22], [23], supporting the model prediction of lactate production in Phase 3. The empirical evidence indicates that healthy, mature, non-starved brains take up ammonia [24] while the brains of Alzheimer patients release ammonia [24], [25], supporting the model prediction of a transition from uptake (Phase 1) to excretion of ammonia (Phase 2 and 3).

The *in silico* predictions are also a motivation for further experimental work. According to our analysis the switch from ammonia uptake to ammonia excretion in Phase 2 precedes the switch from lactate uptake to lactate excretion in Phase 3. This prediction points to measurements of ammonia levels as an earlier indicator of neurodegeneration than lactate levels. It also remains to be verified whether the neurons follow the metabolic switches Phase 1 → Phase 2 → Phase 3 as the neuronal activity is increased.

## Materials and Methods

### Model description

As starting point, we utilize a genome-scale metabolic reconstruction of a generic human cell [27] that includes most biochemical reactions catalyzed by enzymes encoded in the human genome. We add auxiliary reactions to represent nutrient uptake, excretion of metabolic byproducts, basal ATP demand needed for cell maintenance, basal rate of protein degradation, basal rate of RNA degradation, and synthesis of cell biomass components (proteins, lipids, RNA and DNA) (Table S1 in [26],). We assume that the cell is in a steady state where the production and consumption of every metabolite and macromolecules balances, known as the flux balance constraint [27]. We use *S_mi_* to denote the stoichiometric coefficient of metabolite *m* in reaction *i*. We use *f_i_* to denote the steady state reaction rate (flux) of the *i* ^th^ reaction in the metabolic network, where all reversible reactions are represented by a forward and backward rate, respectively. Reactions are divided into nutrient import reactions (*RI*), reactions taking place outside the mitochondria (*RnM*) and reactions taking place in the mitochondria (*RM*). We use *φ_c_* to denote the relative cell volume fraction occupied by the *c* ^th^ cellular compartment, where a compartment represents the overall contribution of macromolecules of certain type (e.g., ribosomes) or of certain cell organelle (e.g., mitochondria). Specifically, here we consider proteins that do not form part of enzyme complexes or ribosomes (*P0*), all metabolic enzymes catalyzing reactions outside the mitochondria (*EnM*), all metabolic enzymes catalyzing reactions in the mitochondria (*EM*), ribosomes (*R*), and mitochondria (*M*). We assume the energy demand *f_ATP_* and the total relative volume fraction occupied by macromolecules and organelles (*φ_max_*) are known and are given as input parameters of the model. Finally, we estimate the metabolic fluxes and compartment densities as the solution of the following optimization problem:

Find the *f_i_* and *φ_c_* that minimize the sum of nutrient import costs 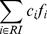
(1) subject to the metabolic constraints: flux balance constraints 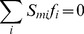
(2) minimum/maximum flux constraints 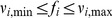
(3) minimum/maximum volume fraction constraints 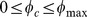
(4) molecular crowding constraints
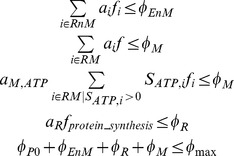
(5) where *c_i_* is the nutrient import cost associated with the uptake reaction *i*, *a_i_* = *v_i_*/*k_eff,i_* are the crowding coefficients of metabolic enzymes (enzyme molar volume/enzyme effective turnover)[34], *a_R_* = *v_R_*/*k_R_* is the ribosome crowding coefficient (ribosome molar volume/protein synthesis rate per ribosome), and *a_M,ATP_* = *v_s,M_*/*r_M_* the crowding coefficient of mitochondria ATP generation (ATP synthesis rate per mitochondria mass/mitochondria specific volume) [35], [36].

### Nutrient import costs

We assume that the cost of importing molecules is proportional to their size and use the molecular weight as a surrogate for size. Within this approximation the import cost *c_i_* is equal to the molecular weight of the molecule imported in the auxiliary uptake reaction *i* (Table S1 in [26]).

### Kinetic parameters

The effective turnover numbers *k_eff,i_*, quantify the reaction rate per enzyme molecule. For example, for an irreversible single substrate reaction satisfying Michaelis-Menten kinetics, *k_eff_* = *kS*/(*K*+*S*), where *k* is the enzyme turnover number, *K* the half-saturation concentration and *S* the substrate concentration. The turnover numbers of some human enzymes are reported in the BRENDA database [37], February 2010 release. More precisely, the BRENDA database was downloaded and parsed for human enzymes with reported turnover numbers. For some enzymes more than one turnover value was reported and in such cases we took the average between them. They have a typical value of 10 sec^−1^ and a significant variation from 1 to 100 sec^−1^ (Table S1). However, for most reactions we do not know the turnover number, the kinetic model, or the metabolite concentrations, impeding us to estimate *k_eff_*. To cope with this indeterminacy we performed a sampling strategy, whereby the *k_eff,i_* were sampled from a reasonable range of values, and then focused on the predicted average behavior and 90% confidence intervals (see Sensitivity analysis below).

### Crowding coefficients

Dividing the mitochondrium specific volume (3.15 mL/g in mammalian liver [38] and 2.6 mL/g in muscle [39]) by the rate of ATP production per mitochondrial mass (0.1–1.0 mmol ATP/min/g [40], [41], [42]) we obtain *a_M_* values between 0.0026 to 0.032 min/mM. Except when specified, we use the median 0.017 min/mM. Dividing the ribosome molar volume (*v_R_* = 4,000 nm^3^×6.02 10^23^/mol = 2.4 L/mmol) by the rate of protein synthesis per ribosome (0.67 proteins/min [43]) we obtain *a_R_* = 3.6 min/mM. The enzyme crowding coefficients were estimated as *a_i_* = *v_E_*/*k_i_*. Multiplying the average molecular weight of human enzymes (10,6843 g/mol) by the enzymes specific volume (approximated by the specific volume of spherical proteins, 0.79 mL/g [44]) we obtain an estimated enzymes molar volume of *v_E_* = 0.084 L/mmol.

### Macromolecular composition

Proteins were divided into three pools: ribosomal-, components of metabolic enzyme complexes-, and non-metabolic proteins. Each ribosome contributes to *n_PR_* = 82 proteins/ribosome (49 in the 60S and 33 in the 40S subunits [45]). The ribosomal protein concentration was computed as *P_R_* = *n_PR_φ_R_/v_R_*. Each enzyme contributes with *n_PE_* = 2.6 proteins in average, estimated as the average enzyme molecular weight (10,6843 g/mol, reported above) divided by the median molecular weight of a human protein (40,835 g/mol). The median molecular weight of a human protein was estimated from the median protein length (355 amino acids [46]) and the typical amino acid composition [31]. The enzyme related protein concentration was computed as *P_E_* = Σ*_i_n_PE_f_i_/k_i_*. The concentration of non-metabolic proteins was estimated as 85% (10% metabolic enzymes and 5% ribosomal protein [46]) of the reported total protein content per cell dry weight (0.018 mmol/g DW [31]), i.e. 0.015 mmol/g DW. The lipids, DNA and RNA composition were estimated by their relative abundance in a generic mammalian cell [31]. The abundance per cell dry weight were converted to concentrations after dividing by the typical cell specific volume 4.3 mL/g [47]. This resulted in a concentration of non-metabolic protein of *P*_0_ = 3 mM. The maximum macromolecular density of human cells in the absence of osmotic stress is around *φ_max_* = 40% [48].

### Maintenance parameters

The ATP production rate necessary for cell maintenance is 1.55 mmol ATP/g DW/h [31]. The basal protein degradation rate was set to *k_D_* = 0.01/h [49]. The basal rate of RNA degradation was fixed to 0.085 per hour, estimated as the average degradation rate of human mRNAs [50].

### Flux balance for the protein content

The flux balance equation for proteins (equation (2) with *m* = *proteins*) is formulated as follows. We account for three major categories, proteins not associated with metabolism, proteins that are components of enzyme complexes, and ribosomal proteins, with their concentrations (moles/cell volume) denoted by *P*_0_, *P_E_*, and *P_R_*, respectively. In non-proliferating cells, these concentrations will decrease at a rate *k_D_*(*P*_0_+*P_E_*+*P_R_*). The total concentration of proteins in enzyme complexes can be estimated as *P_E_* = *n_PE_E* = *n_PE_*Σ*_i_f_i_*/*k_eff,i_*, where *n_PE_* is the average number of proteins in an enzyme complex (about 2.4) and *E* is the total concentration of metabolic enzymes. Similarly, *P_R_* = *n_PR_φ_R_*/*v_R_*, where *n_PR_* is the number of proteins in a ribosome (82 for the 80S ribosomes) and *φ_R_*/*v_R_* is the concentration of ribosomes. Putting all these elements together, the balance between protein turnover and synthesis implies *f_Protein_sysnthesis_*-*k_D_*[*P*_0_+ *n_PE_*Σ*_i_*(*f_i_*/*k_eff,i_*) + (*n_PR_*/*v_R_*)*φ_R_*] = 0.

### Simulations

The optimization problem in equations (1)–(5) was solved in Matlab, using the linear programming function linprog (File S1). All reversible reactions were represented by an irreversible reaction on each direction with their own effective turnover number *k_eff,i_*. Flux bounds were set to *v_i,min_* = 0 and *v_i,max_* = ∞ unless specified. The lower and upper bounds of the ATP maintenance, basal RNA degradation, and basal protein degradation were set to the maintenance parameters reported above. The lower and upper bounds of the biomass producing reaction were set to zero.

### Sensitivity analysis

The turnover numbers of human enzymes *k* are mostly concentrated in the range from 1 to 100 sec^−1^ (Table S1). Based on this data we sampled the log_10_(*k_eff_*) values from a uniform distribution in the range between log_10_(1) to log_10_(100). For each specified condition we run 100 simulations. On each simulation, for each reaction, a value of *k_eff,i_* is extracted from the distribution described above. With this set of *k_eff,i_* parameters we then solve the optimization problem (1)–(5) and obtain estimates for the reaction rates. Because the *k_eff,i_* are real value random numbers, the solution of the linear optimization problem is unique for each set of kinetic parameters. Based on the 100 simulations we finally estimate the median and 90% confident intervals for the rate of each reaction. 100 simulations were proven to be sufficient to capture the overall range of behavior, since running 1,000 simulations did not result in any significant change of the flux distributions. Specifically, for a given ATP demand and a give reaction, we have performed a Kolmogorov-Smirnov test to determine whether the flux distribution as obtained from 100 kinetic parameter sets is statistical equivalent to that obtained using 1,000 kinetic parameter sets. We performed this test for a protein aggregate density of *P* = 3 mM and different ATP demands. Given that we performed this test for thousands of reactions and to correct for multiple hypothesis tests, we set 0.005 = 0.05/1,000 as the threshold to call statistical significance. In all cases the hypothesis that the distributions are equivalent (*p*>0.005) was satisfied, indicating that the flux distributions obtained for 100 kinetic parameter sets are equivalent o that for 1,000 parameter sets. Therefore, 100 kinetic parameter sets is sufficient to have a reasonable exploration of the flux distribution. The results of this statistical test are reported in the Table S2.

## Supporting Information

Table S1 **Turnover numbers of selected human enzymes.**
(XLS)

Table S2 **Sensitivity analysis.** The metabolic flux predictions are dependent on the choice of kinetic parameters. For each reaction, we determined its flux distribution in 100 kinetic parameter sets or 1,000 parameters sets. This table reports the mean flux of each reaction over 100 kinetic parameter sets and the probability that the flux distribution as obtained using 100 kinetic parameters is different from that using 1,000 kinetic parameters. Values above 0.05 indicate that the null hypothesis is correct, i.e. the distributions are similar and, therefore, 100 kinetic parameter sets is sufficient.
(XLS)

File S1 **Matlab code to run the metabolic model reported here.**
(ZIP)
